# Monitoring biomolecule concentrations in tissue using a wearable droplet microfluidic-based sensor

**DOI:** 10.1038/s41467-019-10401-y

**Published:** 2019-06-21

**Authors:** Adrian M. Nightingale, Chi Leng Leong, Rachel A. Burnish, Sammer-ul Hassan, Yu Zhang, Geraldine F. Clough, Martyn G. Boutelle, David Voegeli, Xize Niu

**Affiliations:** 1 Faculty of Engineering and Physical Sciences, University of Southampton, Southampton, SO17 1BJ UK; 2 Department of Bioengineering, Imperial College London, South Kensington, London, SW7 2AZ UK; 3 Critical Care/ Anaesthesia and Perioperative Medicine Research Unit, University Hospital Southampton NHS Foundation Trust, Tremona Road, Southampton, SO16 6YD UK; 4 Faculty of Medicine, University of Southampton, Southampton, SO17 1BJ UK; 5 Faculty of Environmental and Life Sciences, University of Southampton, Southampton, SO17 1BJ UK; 6 Present Address: Now at Department of Sport, Exercise & Health, University of Winchester, Winchester, SO22 4NR UK

**Keywords:** Fluid dynamics, Lab-on-a-chip, Sensors

## Abstract

Knowing how biomarker levels vary within biological fluids over time can produce valuable insight into tissue physiology and pathology, and could inform personalised clinical treatment. We describe here a wearable sensor for monitoring biomolecule levels that combines continuous fluid sampling with *in situ* analysis using wet-chemical assays (with the specific assay interchangeable depending on the target biomolecule). The microfluidic device employs a droplet flow regime to maximise the temporal response of the device, using a screw-driven push-pull peristaltic micropump to robustly produce nanolitre-sized droplets. The fully integrated sensor is contained within a small (palm-sized) footprint, is fully autonomous, and features high measurement frequency (a measurement every few seconds) meaning deviations from steady-state levels are quickly detected. We demonstrate how the sensor can track perturbed glucose and lactate levels in dermal tissue with results in close agreement with standard off-line analysis and consistent with changes in peripheral blood levels.

## Abstract (web summary)

Continuous real-time measurement of biomarker levels in body fluids offers many exciting possibilities. Here, the authors develop an integrated wearable droplet microfluidic sensor that combines continuous sampling of tissue fluid with in situ analysis using wet-chemical assays.

## Introduction

Chemical signalling changes continuously in the human body. Biomarker concentrations rise and fall in complex patterns and on different time scales: from hourly to daily fluctuations of metabolites, hormones and inflammatory changes, to millisecond spiking of ions and neurotransmitters at neuronal synapses^1–3^. Continuous measurement of these dynamic chemical signals has significant implications for fundamental physiological science, and can play a crucial role in the development of disease diagnostics, therapeutics, treatments and new drugs^4^.

Recent years have seen the development of continuous wearable technologies for non-invasive monitoring (of sweat, tears, etc.)^5–9^, however, there are questions as to how representative these measurements are of underlying tissue and blood levels. Implanted electrochemical sensors can directly measure tissue biochemistry and have the potential to provide high temporal resolution^10,11^, but foreign body response^12^ and performance drift due to long-term stability of bio-recognition sites (such as immobilised proteins and antibodies) pose great challenges to sensor viability^13,14^. An alternative approach is to develop miniaturised analytical devices based on microfluidics.

Microfluidics allows the miniaturisation of laboratory assays and detection technology to create micro total analysis systems, which have been successfully applied to point-of-care (POC) diagnostics^15–18^. Current POC devices (e.g., lateral flow devices^18^) focus on single-measurement procedures. While accurate and user-friendly, they are manually intensive and the inherent low measurement frequency limits the ability to track dynamic chemical changes. Hence, the application of microfluidics to *continuous* monitoring requires a shift of strategy to autonomous systems that integrate technology for sampling, liquid handling and analysis. This poses challenges as microfluidic systems typically rely on bulky laboratory equipment such as syringe pumps, external valves and microscopes—technology ill-suited for wearable devices.

Here, we present a fully integrated wearable microfluidic sensor, which not only provides accurate, precise and robust fluidic sampling and control, but also in situ chemical assaying using droplets as microreactors. It addresses the challenges of POC monitoring by providing accurate real-time continuous measurement with high temporal resolution in a small wearable package. We demonstrate and validate the device in vivo by using it to monitor perturbed glucose and lactate levels in dermal tissue in healthy volunteers.

## Results

### Device operation

**Fig. 1:**
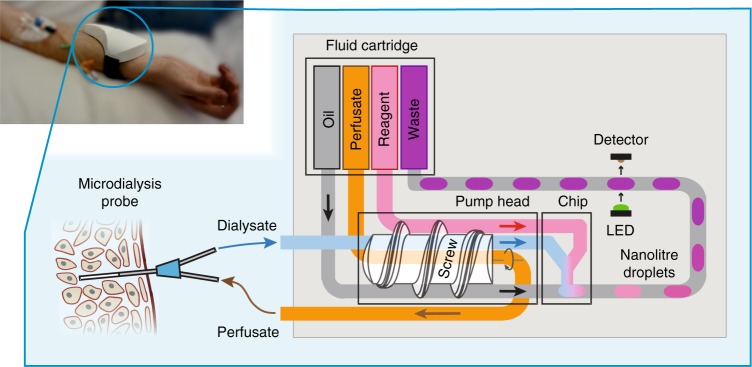
Schematic diagram illustrating the operation of the device. The screw-driven peristaltic pump simultaneously feeds perfusate into a microdialysis probe and withdraws the resulting dialysate into the device. From the pump, dialysate is delivered into a microfluidic chip, where an analyte-specific reagent is added, and the resultant flow immediately segmented into a stream of droplets by the addition of an immiscible oil. Within the droplets, the reagent reacts with the analyte to produce a measurable optical response. The droplets flow out of the chip into low-volume PTFE tubing and downstream to an optical flow cell where the product of the reaction is quantified^31^. A microcontroller (not shown) saves the result to a micro SD card and relays it via Bluetooth to an external device. The analysed droplets are collected in a waste sachet for later disposal. An image illustrating a sensor being used in a clinical setting to monitor tissue is shown top-left

The sensor combines a miniature peristaltic pump, microfluidic chip, optical flow cell, electronics and a fluid reservoir cartridge—all integrated into a small wearable package (Fig. 1, top left), and employs a droplet-flow regime which gives optimal temporal response in microfluidic analysis systems^19–22^. The fluidic architecture can be tailored depending on the analyte and the requirements of its assay. Figure 1 shows the simplest fluidic setup where analysis is via a simple mix-and-read colorimetric assay. The sensor connects to a microdialysis probe (composed of concentrically arranged inlet and outlet tubes connected by a permeable membrane at the probe tip) to allow sampling of interstitial fluid by flowing a sterile perfusate solution (such as phosphate buffered saline, PBS) through the probe. Molecules can freely diffuse from the interstitial fluid into the perfusate at the probe tip, yielding a clean dialysate that is directly representative of the chemical composition of the interstitial fluid. The dialysate is pulled into the sensor, through the pump and then on into a microfluidic chip where it meets an analyte-specific reagent and is subsequently broken into a stream of droplets by introduction of an immiscible oil. Within each droplet the dialysate mixes and reacts with the reagent to generate a coloured product. The concentration of the target analyte determines the strength of the droplet’s colour, which is quantified by an inline optical flow cell. The data from the flow cell is passed to an on-board microcontroller which relays it to an external data-processing device using an integrated Bluetooth transmitter, and also saves it to an SD card.

Traditionally, microdialysis utilises low perfusion flow rates (0.1–5 µL min^−1^) to increase perfusate residence time at the probe membrane and hence achieve high molecular recovery (defined as the fraction of target molecules that diffuse into the perfusate relative to the theoretical maximum). The miniature peristaltic pump in our sensor (illustrated in Fig. 1, detailed design shown in Supplementary Fig. 1) can deliver the required low flow rates while also preventing pressure-driven fluid loss through the membrane by simultaneously pushing and pulling fluid through the probe (see Supplementary Fig. 2, Supplementary Movie 1 and Supplementary Note 1). During the in vivo testing described later, the pump pushed/pulled fluid through the probe at 1 µL min^−1^, which compares well with the 15 µL min^−1^ minimum flow rate of a previously reported wearable microdialysis device^23^. A screw thread is used to drive the fluid along the tubing (Fig. 1) as opposed to the more typical planetary rollers^24^, to allow much slower flow rates at a given motor speed—important for maximising diffusion time across the probe membrane and boosting recovery. The flow rate delivered by each line is controlled by either choosing the inner diameter (ID) of tubing, screw pitch or adjusting motor speed, with average flow rates from 0–20 µL min^−1^ achievable. As the screw turns unidirectionally and continuously compresses the tubing, downstream fluids (oil and reagent) cannot backflow, ensuring only controlled sterile material is introduced to the probe.

**Fig. 2:**
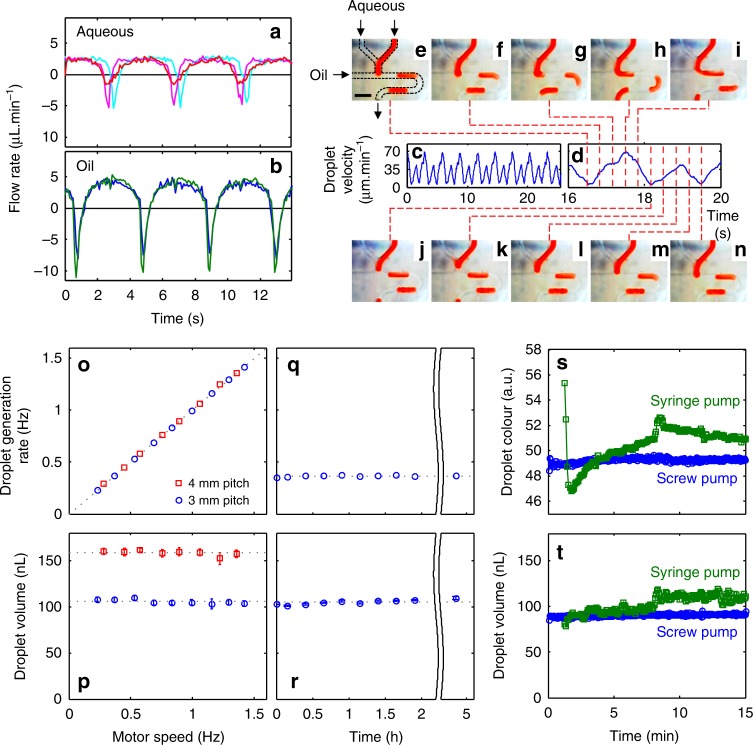
Anti-phase pulsatile flow and its use in droplet generation. **a**, **b** Flow rate over a three-pulse period for three aqueous lines (**a**): perfusate (probe push), dialysate (probe pull) and reagent, and the two oil lines (**b**). **c** Downstream velocity of droplets generated by the pulsatile flows (**a**, **b**) at a T-junction. **d** The linear droplet velocity for a single pumping period composed of one aqueous and oil pulse. The dashed lines correlate the velocity data to droplet generation at the T-junction (**e**–**n**) showing the controlled generation of a single droplet in the single pumping period. Scale bar in (**e**), 1 mm. **o** The droplet generation rate increases linearly with motor speed (and hence flow rate) for both 3 mm (blue circles) and 4 mm (red squares) screw thread pitches. The points fit on a straight line of unity gradient indicating that one droplet is produced in one turn of the pump. **p** Droplet volume is constant with respect to motor speed (and hence total flow rate) with different values for different pitch sizes. Error bars show the standard deviation in droplet size. **q**, **r** Droplet generation rate and volume remain constant when run at constant conditions (0.37 Hz, 3 mm pitch) over several hours. Error bars in (**r**) show the standard deviation in droplet size. **s**, **t** Comparison of the composition and size of droplets produced immediately after start-up using the screw-driven pump with 3 mm pitch (blue circles) and syringe pumps (green squares). The screw-driven pump instantaneously produces droplets of uniform composition (**s**) and volume (**t**), while the syringe pump shows significant drift over time

The pump is key for robust droplet generation. Traditional droplet generation involves coflowing aqueous and oil streams and allowing the interfacial tension to drive the natural formation of droplet flow^25,26^. At low-flow rates and under changeable ambient conditions, this method of droplet formation will be susceptible to perturbation, leading to uncontrolled assay reaction conditions (sample:reagent ratio, reaction time from droplet generation to detection point)^27^. Here, we exploit the pulsatile flow of the peristaltic micropump to reliably produce droplets of uniform volume and sample-to-reagent ratio (ensuring reproducible mixing, reaction stoichiometry, and residence time)^28^. As shown in Fig. 2a, b, the flow profile for each line is characterised by long periods of positive flow (as the turning screw forces fluid along the tubing) interspersed with short periods of negative flow due to the expansion of the tubing as the screw thread disengages (one per turn of the motor). Within the pump, the aqueous lines are positioned together at the top, with the oil lines on the bottom (as illustrated in Fig. 1 and shown in detail in Supplementary Fig. 1) such that the screw thread disengages at different times and produces flow profiles that are out of phase. When fed into a standard microfluidic T-junction the flow imbalances during each pump revolution results in reproducible pulsatile flow (Fig. 2c) that determines how the fluids break into oil-separated aqueous droplets. Within each pumping period (one revolution of screw/pump comprising a single aqueous and oil pulse, Fig. 2d) a transient flow imbalance causes the aqueous phase to be chopped into a droplet at a fixed moment, thus enabling production of a single droplet per turn of the screw thread (as shown in Fig. 2e–n and Supplementary Video 2). As a single turn of the motor gives a single oil pulse and a single aqueous pulse, resulting in the production of a single droplet, the size of the droplet is solely defined by the volume of the aqueous pulse—independent of pump speed and total flow rate. Each aqueous pulse corresponds to the volume of liquid held in the tubing between raised sections of the screw thread, thus droplet volume can be specified by careful choice of tubing diameter and screw pitch.

To demonstrate this we monitored the frequency and size of droplets generated at the T-junction whilst changing the motor speed (Fig. 2o, p) for two different pitches of the screw thread (3 and 4 mm). The droplet generation rate (Fig. 2o) varied linearly with motor speed, with one turn of the motor consistently resulting in the generation of a single droplet irrespective of the pitch. The droplet volume was constant irrespective of changes in flow rate (Fig. 2p), with the longer pitch screw thread producing larger droplets as a result of the increased volume of tubing pinched by the screw thread. Note this droplet generation mechanism requires the aqueous fluid pumped into the main channel of the T-junction to fully block the cross-section of the channel (Fig. 2g, h) before the end of the aqueous oil pulse, otherwise two or more turns may be required to generate a droplet (see Supplementary Fig. 3). Nonetheless, with appropriate choice of pitch and channel size, droplets can be robustly generated with defined volumes and composition, making this an ideal droplet generation mechanism for a field-deployable device. The stability of the droplet generation was assessed by measuring the generation rate (Fig. 2q) and droplet volume (Fig. 2r) over time with a fixed motor speed. Both parameters remain constant over the course of five hours with a 2.0% relative standard deviation (RSD) and even over multiple days (Supplementary Fig. 4, 2.2% RSD) indicating that the pump can be used to reproducibly sample and generate droplets over prolonged periods.

The microfluidic chip was mounted directly on the pump (see Supplementary Fig. 1d) to both minimise sample dispersion prior to droplet generation^29^, and reduce flow stabilisation time. Due to the minimisation of the fluidic volume we found our screw pump could generate droplets immediately after startup with uniform composition (Fig. 2s, blue circles, 49.3 ± 0.2 a.u.) and size (Fig. 2t, blue circles, 91 ± 1.3 nL). This is notable when compared to droplets generated using the conventional method of coflowing fluids from syringe pumps (Fig. 2s, t green squares) where it took over a minute (74 s) to start producing droplets and, once stabilised, exhibited greater variance (e.g., volume 110 ± 3.5 nL, after 9 min stabilisation time, Fig. 2t). The improved stability and negligible ramp-up time of the screw pump shows how the anti-phase peristaltic droplet generation method is highly robust and suitable for supporting a plug-and-play sensor device.

### Glucose assay implementation

**Fig. 3:**
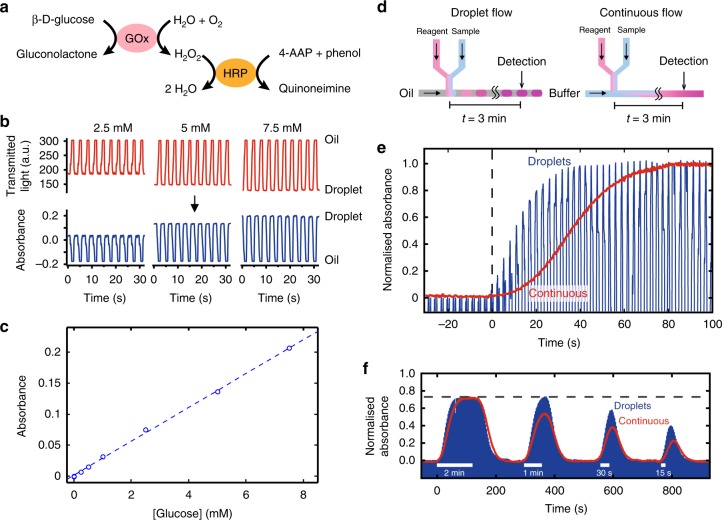
Glucose assay and sensor calibration. **a** The reaction mechanism for the glucose assay. Glucose is broken down by glucose oxidase (GOx) to yield gluconolactone and hydrogen peroxide (H_2_O_2_), which is then catalysed by horseradish peroxidase (HRP) in the presence of phenol and 4-aminoantipyrine (4-AAP) to form a red-violet coloured product quinoneimine. **b** Examples of raw data from the flow cell (top) converted to absorbance (bottom) using a pre-recorded blank measurement of droplets with 0 mM sample. **c** The measured absorbance was linear with respect to glucose concentration up to 8 mM. **d** Flow schematic diagram for droplet flow (left) and continuous flow (right) experiments. **e** Comparison of the sensor’s temporal response to a concentration step change when operating in droplets (blue) and continuous flow (red). In droplet flow, it took 38 s, or 13 droplets, to reach the step plateau whilst the continuous stream took 80 s. **f** Comparison of dynamic in vitro microdialysis response when operating in droplets (blue) and continuous flow (red). For microdialysis sampling, we imposed concentration change at the probe of varying durations, from 2 min to 30 s, to simulate transient events in tissues. The response is normalised relative to the response obtained when the glucose solution was directly aspirated, bypassing the microdialysis probe. Hence, the dotted line represents the relative recovery of the microdialysis probe

In applying the sensor to in vivo monitoring of tissue chemistry, we chose first to monitor glucose as an exemplar analyte. Glucose is ubiquitous within bodily tissues, has well-understood pharmacokinetics, is routinely measured in a clinical setting, and as such was deemed highly appropriate for validating the sensor. To measure glucose, we implemented a colorimetric assay based on the Trinder reaction^30^ (see Fig. 3a and Methods) due to its sensitivity and robustness. For this assay all reactants are premixed into a single solution, which is introduced to the dialysate (see Fig. 1) and reacts to yield a red/purple coloured product which is quantified by a downstream flow cell^31^ as an absorbance measurement (Fig. 3b). With a light path-length of 0.5 mm, the assay gaves a linear absorbance response up to at least 8 mM (Fig. 3c), with a sensitivity of 0.026 ± 0.002 mM^−1^ (*n* = 6) and a limit of detection of 68 µM (defined as three times the standard deviation of a series of blanks). While tissue concentrations in diabetic patients can reach up to 20 mM (360 mg dL^−1^), in vivo recoveries for microdialysis probes are typically <40%^32^ meaning that we would not expect dialysate concentrations greater than 8 mM (144 mg dL^−1^).

Droplet-flow removes Taylor dispersion^33^, thus improving temporal resolution relative to continuous (single-phase) flow. To quantify the improvements here, we compared the temporal response of an imposed concentration step-change using first droplet flow and then continuous flow regimes. We first tested the sensor without attaching to a microdialysis probe. For the droplet-flow experiment, the device operated as previously described while for continuous flow the oil was replaced with phosphate buffer (see Fig. 3d) to ensure the same total flow rate. The results, shown in Fig. 3e, were normalised and time aligned to the start of the step change so that the temporal response of each flow regime could be easily compared.

In both cases, the absorbance increased from zero in the absence of glucose, to a maximum corresponding to the introduction of a 5 mM (90 mg dL^−1^) glucose solution. The continuous flow showed a gradual 80 s transition due to the effects of Taylor dispersion blurring the composition of the two concentrations as they travelled through the system. The dispersion was also seen in the droplet-flow system due to the unavoidable continuous flow between the inlet and droplet generation (approximately 60 mm channel length). Nonetheless, in droplet flow the transition was much quicker, taking less than half the time to reach maximum (38 s versus 80 s) and less than a third of the time to get to 50% of maximum (10 s versus 35 s).

The temporal response under dynamic tissue conditions was then simulated using an in vitro microdialysis experiment. A concentric microdialysis probe (fabricated in-house, 13 kDa molecular weight cut off, MWCO) was attached to the pump, perfused with PBS and then immersed in a vigorously stirred 5 mM (90 mg dL^−1^) glucose solution for a set period varying between 2 min and 15 s (Fig. 3f). For the 2 min immersion, both the continuous and droplet flow showed a similar response, with the absorbance rising, plateauing and then returning to the starting value. The plateau value was 75% of the value obtained when the solution was directly aspirated, and corresponds to the in vitro probe relative recovery. This value compares favourably to the 10% previously reported for an analogous wearable microdialysis device^23^. The introduction of the probe and hence an increased length of continuous flow before droplet generation caused a general increase in response times, however, the droplet-phase experiment was still markedly quicker than the continuous experiment, with the signal rising in 68 s versus 100 s and falling in 69 s versus 103 s. For the 1 min immersion, the continuous flow operation failed to reach the peak value obtained from the longer immersion (dashed horizontal line) due to the falling trend overlapping with the rising trend. The droplet-flow setup showed an increase response due to reduced dispersion however, and gave a peak response at the expected measured sample concentration value. As the immersion time decreased below 1 min, the peak measured sample concentration decreased for both the droplet and continuous flows as expected, however, the signal from the droplet flow was consistently higher by 0.18 absorbance units, equivalent to an improvement of 34% (for 60 s pulse), 51% (30 s) and 72% (15 s), over continuous flow.

The small overall size of the device allows a short connection (~7 cm) from the microdialysis probe to the measurement point, making it possible to capture transient events as short as 15 s even with the continuous fluidics approach. Using droplet-flow however, shortens the response time, better preserves the sample concentration characteristics and improves the device’s ability to capture fast transient concentration changes.

### In vivo glucose monitoring

To validate the sensor, it was used to monitor dermal interstitial glucose levels of five healthy human volunteers subjected to an oral glucose tolerance test (see Methods). At the start of the experiment, glucose levels were monitored continuously for approximately 60 min to establish a baseline. A challenge of 50 g of glucose in ~100 ml water was then administered orally, and the glucose levels tracked over time for up to 180 min. To contextualise and validate the sensor results, the tissue was also sampled and analysed using standard microdialysis and manual offline analysis (see Methods), and venous blood concentrations were periodically measured.

**Fig. 4:**
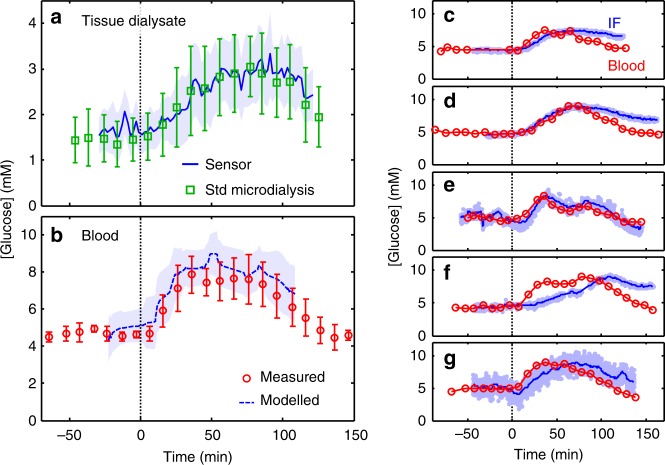
In vivo measurement of glucose in subcutaneous interstitial fluid. **a** Average (*n* = 5) dialysate glucose measured by the sensor (blue line and shaded area) and standard microdialysis sampling and offline analysis (green squares and error bars). Each is shown as a function of time since oral glucose administration (vertical dashed line, *t* = 0) and the error bars/area represent the standard deviation from subject-to-subject variation. **b** Mean (*n* = 5) venous blood glucose (red circles and error bars) shown as a function of time since oral glucose administration (vertical dashed line, *t* = 0). The error bars represent the standard deviation from subject-to-subject variation. The blue dashed line indicates the blood glucose predicted from the sensor data calculated using a two-compartment model^37,38^ (see Methods and Supplementary Fig. 14). The shaded blue area show the standard deviation of the model output, while the dashed blue is a running average (median ± 20 min). **c**–**g** Plots showing the blood glucose (red circles) and estimated interstitial fluid glucose (IF, light blue points with blue line showing a rolling average) for each individual

The average dialysate (tissue) glucose responses over time as measured by the sensor (blue line) are shown in Fig. 4a, with the pale blue surrounding area indicating the subject-to-subject variation. Following the administration of the oral glucose (*t* = 0, marked by the vertical dashed line), glucose levels increased from an initial baseline of 1.7 mM (31 mg dL^−1^) to a maximum of 2.9 mM (52 mg dL^−1^) over the course of 60 min, plateauing, then falling after 90 min The standard microdialysis (green squares) constitutes the nearest equivalent standard sampling/analysis method to that used by the sensor. Importantly, both the sensor and standard microdialysis results closely agreed in both the absolute values and the variation over time.

In the blood (Fig. 4b, red circles) glucose levels rose from a baseline of 4.5 mM (81 mg dL^−1^) to 7.8 mM (141 mg dL^−1^) after 45 min, plateauing then falling after 75 min and returning to baseline. The blood glucose levels were typically two to three times higher than the dialysate values, reflecting both the different concentrations in the different compartments and the fractional recovery across the semipermeable membrane of the microdialysis probe which is universally found to be reduced in in vivo experimentation^32^ (see ESI for further discussion). The earlier rise and fall in blood glucose levels relative to the interstitial fluid reflects the physiological pathway of the orally administered glucose, first absorbed into the blood stream before subsequent distribution to tissue via the capillary network, as previously observed^34–36^. This pathway has been previously modelled by Stein et al. using a two-compartment pharmacokinetic system^37,38^. Using this model we found that the sensor data could accurately predict the variation in blood glucose levels, as shown by the blue dashed line in Fig. 4b.

Figure 4c–g shows the variation of blood glucose and device-measured dialysate glucose as a function of time for each individual subject (the accompanying standard microdialysis results are shown in Supplementary Figs. 5 and 6 and discussed in Supplementary Note 2). The sensor results were then used to estimate interstitial fluid concentrations using a two-point calibration method^38,39^ (see Methods), which allows easier comparison of the relative changes in blood and tissue concentrations. While the magnitude and speed of the blood glucose levels varied, the increase and fall of sensor data consistently followed it, with lag times varying on a subject-to-subject basis from a few minutes (e.g., Fig. 4e) to approximately half an hour (e.g., Fig. 4f) but all within the expected range^37^. In all cases, the tissue dialysate glucose levels showed the expected trends and tracked the blood glucose levels, demonstrating that our device is capable of resolving real-time physiological dynamics in vivo.

### Lactate assay implementation

To demonstrate that different analytes can be measured by the sensor, we then targeted lactate for in vivo monitoring. Lactate, like glucose, is an important small molecule involved in metabolism and is a crucial diagnostic indicator for a range of patho/physiological conditions, such as in transplant surgery^40^, brain injury^41,42^, and sport science^43^.

**Fig. 5:**
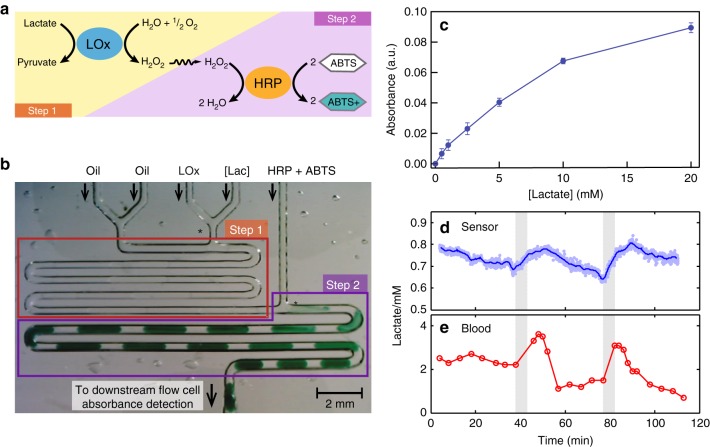
Lactate assay, calibration, and in vivo testing. **a** Reaction scheme for the lactate assay. Step 1—Lactate is broken down by lactate oxidase (GOx) to yield hydrogen peroxide (H_2_O_2_) and pyruvate. Step 2—The H_2_O_2_ is then catalysed by horseradish peroxidase (HRP) to oxidise 2,2′-azino-bis(3-ethylbenzothiazoline-6-sulfonic acid) (ABTS) to a blue-green coloured species (ABTS^+^). **b** Image of the lactate assay being performed on-chip. The asterisks (*) denote T junctions. Transparent droplets containing lactate samples and LOx at a 1:1 ratio are generated at the T-junction in Step 1. They then travel through the serpentine channel with enough time for the LOx reaction to run to completion. Upon dosing with HRP/ABTS at the second T-junction in Step 2, an intense blue-green colour starts to develop. **c** The two-step lactate assay (blue circles) gives a monotonic increase to 20 mM, with a near linear response at lower concentrations. Error bars refer to the standard deviation from measurement of multiple droplets. **d** In vivo sensor measurement of dialysate lactate from subcutaneous interstitial fluid. Each data point represents a measurement from a single droplet with the line indicating the running average. The shaded areas indicate periods of blood occlusion to the tissue. **e** Blood lactate during the same period

We employed a solution-based colorimetric lactate assay^44^ analogous to the glucose assay (Fig. 5a), but which needed to be performed in two steps as the high turnover of lactate oxidase^45,46^ (LOx) can lead to futile mediation^47^ (whereby local oxygen levels are depleted and the chromophore from step 2 is then consumed instead) if performed as a one-step assay. Accordingly, a microfluidic chip was designed and fabricated in poly-di-methyl-siloxane (PDMS) (Fig. 5b), and the pump modified to accommodate another aqueous line (see Supplementary Fig. 7). In the first reaction step, LOx reagent is added to the sample stream at a volumetric ratio of 1:1 and the coflow immediately broken up into discrete droplets at a T-junction (Fig. 5b). The droplets travel through a serpentine channel with enough reaction time for the LOx reaction to run to completion. In reaction step 2, the droplets are dosed with a second reagent, triggering the conversion of the H_2_O_2_ (produced in step 1) into H_2_O and the oxidation of the chromophore, causing the droplets to develop an intense blue-green colour as shown in Fig. 5b and Supplementary Movie 3. This method could measure lactate from 0.5 to 20 mM (Fig. 5c) with a quasi-linear response with sensitivity of 0.1 ± 0.002 mM^−1^ (*n* = 6) at concentrations below ~1.5 mM and a limit of detection of 17 μM (*n* = 3).

### In vivo lactate monitoring

To validate the sensor’s ability to monitor lactate in vivo, we tracked the variation of lactate in the dermal tissue of the forearm of healthy human volunteers subjected to mild tissue ischaemia. Ischaemia was induced using a standard blood pressure cuff periodically inflated to suprasystolic pressure to restrict blood flow to the arm (see Methods).

Example results from one individual are shown in Fig. 5. Dialysate lactate as measured by the device (Fig. 5d) decreased slightly from 0.8 to 0.7 mM during an initial baseline period (*t* = 0–38 min). When the cuff restricted blood flow to the arm (grey shaded area, *t* = 38–43 min) lactate levels increasing by 0.05 mM (7%) in 5 min, consistent with the generation of lactate from tissue under hypoxic conditions^48^. After the cuff was released and blood flow restored to the arm, lactate levels increased at a slower rate for another 7 min before returning to a gentle decrease, as seen during the baseline period. The effect of the vascular occlusion was also evident in the blood, with blood lactate levels spiking and remaining elevated for ten minutes post-occlusion as the accumulated tissue lactate was cleared, before dropping to a new baseline level. Similar trends were observed during a second cuff inflation (*t* = 77–82 min): The blood lactate was elevated in the period immediately following occlusion, while the dialysate lactate again rose sharply during the occlusion (~0.10 mM, 14%) and continued to increase, at a slower rate, in the 7 min immediately following. While the magnitude of the response varied between different individuals (*n* = 4, see Supplementary Fig. 8 and Supplementary Note 3), the trends were consistent, with occlusion leading to an increase in lactate levels and a subsequent short-lived peak in blood lactate. Throughout, the dialysate lactate measured by standard microdialysis and offline analysis was in agreement with the sensor data (Supplementary Fig. 9) as previously seen for glucose measurement.

The relative changes in analyte levels here were much smaller than those seen in the oral glucose tolerance test and occurred over shorter timescales. Whereas dialysate glucose levels doubled or even tripled over 40–120 min depending on individual subject response (see Fig. 4c–g), lactate levels exhibited total increases of 13 and 25% in each 7 min period (Fig. 5d). Nonetheless, the device could still clearly and accurately describe the changes in interstitial lactate levels, easily discerning finer details such as the two distinct periods of lactate increase during and immediately following vascular occlusion.

## Discussion

In this paper, we have shown a wearable droplet microfluidic-based device for continuous real-time sampling and measurement of tissue biochemistry, with the use of droplet flow resulting in much improved temporal resolution. Traditionally, droplets are generated by coflowing liquids at a T- or flow-focussing junction, and rely on highly stable flow delivered by bulky high-end syringe or pressure-driven pumps. Here the unique anti-phase peristaltic pumping method of droplet generation meant droplets could be robustly generated using a single miniaturised pump and hence a small overall device. Consequently the sensor can be placed in close proximity to the sampling site to minimise lag time and reduce the smearing effect of Taylor dispersion prior to droplet generation. The creation of a robust method to generate droplets raises the possibility of other droplet-based processes (for example, digital PCR) moving out of the laboratory and into the clinic for point-of-care testing^49^.

The versatility of the sensor is highlighted by the fact that the same basic platform can accommodate different assays. Here, we implemented a single-step mix-and-read assay for glucose measurement and a more complex two-step assay for lactate. However, it will be possible to implement a variety of assays to detect different biomarkers as a range of different chemical operations are possible in droplet-based systems (e.g., droplet fusion^50–52^, splitting, dosing, gel-droplet rheological transformation^53^ and movement of analytes between droplets on magnetic beads^54,55^). Likewise, the modular nature of the device means other analytical detection methods apart from absorbance could be used^56^ such as fluorescence^57^, chemiluminescence^58,59^, electrochemical^43^ and separation techniques^60^. In addition, we note that the microdialysis probe used to sample interstitial fluid is not intrinsic to the device, and the sensor could alternatively sample and analyse fluids directly. Inline quality assurance measures could also be implemented. Droplet size and velocity could be optically determined to monitor and report any deviations in flow conditions^61,62^, and as a single pump can drive multiple fluidic streams, droplets of blanks and standards could be periodically introduced to continuously calibrate the sensor and track any changes in sensitivity over time^63^.

The small size and autonomous operation of the sensor make it highly practical. During in vivo testing the offline blood and standard microdialysis measurements used to benchmark the device required near-constant manual operation. Indeed, during lactate testing a minimum of two people were needed to achieve the required sampling rate post-occlusion (a measurement every two minutes). The sensor, however, did not require any human interaction after set-up, giving a live feedback of analyte levels in the dialysate stream with a measurement every three seconds. The wearability of the device also allows continuous measurement of mobile subjects. Indeed, during in vivo glucose testing, there were two separate instances where participants requiring toilet breaks visited the restroom with the sensor still attached and running.

We envisage that our device is broadly applicable to any scenario where chemical composition needs to be continuously monitored in situ and in near realtime. The current greater size and complexity of our device relative to embedded electrochemical sensors mean that it is less appropriate for use in continuous glucose monitoring (CGM), where the sensor must be accommodated within the active lifestyle of an otherwise-healthy diabetic. However, the autonomy and versatility of the proposed device make it an attractive technology for clinical settings (intensive care, trauma units, emergency medicine, etc.) where accurate and real-time monitoring of key biomarkers is desirable to minimise decision-making time and enable rapid clinical responses. The small, wearable nature of the sensor will be advantageous in high dependency units, where working space for clinicians around a patient is often cluttered with a variety of monitoring and fluid administration equipment. Likewise, the device could be used to monitor in vivo concentrations of therapeutic drugs or characterise the pharmacokinetics of new drugs, and it will be of interest to sport scientists and other researchers looking to monitor fundamental physiological processes in the body. When combined with physical sensors, such as electrocardiograms, accelerometers or advanced imaging, a wide spectrum of physical properties and biochemical data can be measured simultaneously and be cross-correlated. This wealth of high quality and multi-mode data will be instrumental for the development of precision and personalised medicine and healthcare.

## Methods

### Chemicals and reagents

For the glucose assay d-glucose, glucose oxidase, horseradish peroxidase (HRP), 4-aminoantipyrine (4-AAP) and phenol were purchased from Sigma-Aldrich (Dorset, UK) and used without further purification. For the lactate assay LOx and HRP were purchased from Sekisui Diagnostics (UK) and used without further purification. 2,2′-azino-bis(3-ethylbenzothiazoline-6-sulfonic acid) diammonium salt (ABTS) was purchased from Sigma-Aldrich (Dorset, UK) and used without further purification. Deionised water (18.2 MΩ cm, MilliQ) was used throughout. Phosphate buffer (PB) at 0.1 M was prepared from sodium phosphate dibasic and sodium phosphate monobasic salts, and adjusted to pH 7.0. The carrier oil used in all droplet experiments was FC-40 (Fluorinert, 3M, MN, USA), with an non-ionic tri-block copolymer surfactant synthesised in-house^64^ at loadings of 1.5 and 0.4 wt% for the glucose and lactate work, respectively.

The single reagent for the glucose assay consisted of 6.25 mM 4-AAP, 18.75 mM phenol, 22.5 U mL^−1^ HRP and 45 U mL^−1^ GOx in PB. The reagent was freshly made from stock solutions of the constituent reactants prior to the experiment, and was covered to avoid exposure to ambient light. Glucose calibration standards were made up in 0.1 M PB and frozen until required.

The first reagent for the lactate assay consisted of 60 U mL^−1^ LOx dissolved in PB. The second reagent consisted of 100 U mL^−1^ of HRP and approximately 100 mM ABTS prepared in PB. For the one-step tests, the homogeneous reagent consisted of 60 U mL^−1^ of LOx, 100 U mL^−1^ of HRP and approximately 100 mM ABTS in PB. Lactate calibration standards were made up in 0.1 M PB and frozen until required.

### Microdialysis probe

Concentric microdialysis probes fabricated in-house were used throughout this paper. The microdialysis probe were made of a semi-permeable tubular membrane (ID 200 µm and OD 280 µm; Spectra/Por^®^ in vivo microdialysis hollow fibres, Spectrum Labs, CA, USA) of 20 mm length, with a characterised MWCO of 13 kDa, ensuring large molecules did not pass through the membrane into the dialysate stream. One end of the membrane fibre was plugged while the other end was joined to a shaft made of fused silica tube (ID 250 µm, OD 360 µm, length 15 mm; Polymicro Technologies, AZ, USA) for mechanical support during implantation of the probe into tissues. The inlet of the probe was made of polyethylene tubing (ID 0.38 mm, OD 1.09 mm; Portex^®^ Smiths Medical, UK) and joined to the shaft. To minimise the fluidic volume after the membrane, a fine-bore fused silica capillary (ID 75 µm, OD 150 µm; Polymicro Technologies) was used as the outlet of the probe. The length of the inlet and outlets was 11 cm measured from the tip of the probe. For in vivo applications, the microdialysis probes were sterilised by ethylene oxide (Meridian Medical, UK) and perfused with sterile PBS solution (pH 7.4, Tayside Pharmaceuticals, UK) before use.

### Pump head

The pump head was composed of a series of parallel poly-vinyl chloride (PVC) tubing (wall 500 µm, ID 190 or 250 µm for aqueous or oil lines, respectively, Gradko International Ltd., UK) positioned concentrically around a custom fabricated metal screw, as illustrated in Supplementary Fig. 1 (glucose) and Supplementary Fig. 7 (lactate). The screw was turned by a DC motor (Pololu 298:1 Micro Metal Gearmotor HP) held in place by a 3D-printed frame (VeroClear, Objet500 Connex3). The tubing was glued (Loctite 4062) onto two 3D-printed supports (VeroClear, Objet500 Connex3) corresponding to the oil and aqueous lines. The supports sat above and below the screw, and were held in place by a 13 mm diameter tool clip (81050, Springmasters, UK) which compressed the tubing against the screw.

To enable easy attachment of the microfluidic chip directly on to the pump head, the tubing exiting the pump head was cut so as to be flush with the 3D-printed structures. Totally, 6 mm lengths of 27-gauge stainless steel tubing were inserted into each exit hole so that ~3mm protruded from the pump head. The protruding section was then covered with PVC tubing (OD 1 mm) to make the protruding tubing wider and better fit the punched inlet holes of the microfluidic chip.

### Microfluidic chip

Droplets were generated in a PDMS microfluidic chip cast from a 3D-printed mould. The mould was designed in 3D CAD software (SolidWorks, Dassault Systemes) with nominal channel dimensions of 100 µm width and 600 µm height. To allow PTFE tubing to be inserted to take droplets off-chip, the end of the main channel featured an enlarged section (cross-section 500 µm width, 550 µm height) at its terminus (see Supplementary Fig. S10a, b). Both the glucose (Supplementary Fig. 10a) and lactate (Fig. 5b, Supplementary Fig. 10b) chip designs featured two oil inlets. The extra oil line was included to increase the total flow rate of the system and reduce the amount of time each droplet took to travel to the flow cell. The chip mould was printed in VeroClear material using an Objet500 Connex3 polyjet printer (Stanford Marsh Ltd.). After printing, the mould was left in an oven overnight at 70 °C to remove any remaining volatile precursor materials. Polydimethylsiloxane (PDMS Sylgard 184) was made up at a ratio of 10:1 elastomer:curing agent, poured into the mould, degassed under vacuum, and then cured at 70 °C for at least 1 h. After removal from the mould, access holes for the inlets were punched using a 1 mm biopsy punch and the microfluidic channels sealed by the half-cure method^65–67^ in which the channels were bonded to a semi-cured flat piece of PDMS. The chips were then cut to shape, ensuring that the end of the main channel was open to allow insertion of PTFE tubing.

To allow reliable droplet generation, the channels were treated to ensure preferential wetting by the fluorous oil. This was initially done by filling the channels with Aquapel (PPG Industries) using a syringe and blunt dispensing needle, waiting for approximately 5 min, and then flushing the channels with air. The chips were then left overnight in an oven at 70 °C. For later work (lactate testing) we moved to surface treating the channels using Novec 1720 (3M). Here, the channels were filled with Novec 1720 using a syringe and blunt dispensing needle and allowed to evaporate (~10 min) under ambient conditions. The chips were then heated using a hotplate at 130 °C for 15 min and then left overnight in an oven at 70 °C.

So that droplets could reliably exit the chip into tubing, a robust user-friendly connector (see Supplementary Fig. 10) was fabricated and attached to the chip as follows. A short length of PTFE tubing (ID 0.4 mm, OD 0.7 mm, Adtech Polymer Engineering, UK) approximately 4 mm in length was half-inserted into a 5 mm length of silicone tubing (0.5 mm ID, 3.6 mm OD, RS Components, UK) such that 2 mm still protruded. The protruding section of PTFE tubing was then inserted into the chip, such that the silicone tubing was then flush with the side of the chip. The silicone tubing was bonded to the chip by addition of a small quantity of PDMS at the join and cured in an oven at 70 °C overnight. Once cured, PTFE tubing could be repeatedly inserted into, and removed from, the silicone connector without any risk of damaging the chip itself. Images of finished chips are shown in Supplementary Fig. 10c, d.

### Flow cell design and setup

The flow cell, shown in Supplementary Fig. 11, was based on a previously reported design^31^ and consisted of a black acrylic body which held the optical components and directed the light path through the fluid as it passed through PTFE tubing. The body was micromilled using a LPKF Protomat S100 micromill (LPKF Laser & Electronics Ltd., Berkshire, UK) from two 3 mm-thick black polymethylmethacrylate (PMMA) blocks. A straight centre groove of dimension 1.0 mm by 1.0 mm was milled to take the PTFE tubing. Perpendicular to the groove, a through-hole of square cross-section (0.4 mm) was milled to direct the light path. Recesses at each end of the through-hole were milled to hold an light emitting diode (LED) (516 nm for the glucose assay, 637 nm for the lactate assay, Avago Technologies, USA) and a light-to-voltage converter (TSL257, Texas Advanced Optoelectronic Solutions, USA). The two PMMA pieces were glued (Loctite, Hertfordshire, UK) together with the tubing inserted into the channels.

### Electronics and data management

A 1.5 V AA battery was used to power the motor, whilst a separate 1.5 V AA battery was used to power all electronics via an OEM DC–DC step-up module which outputted a 5 V supply. A PCB fabricated in-house was used to distribute the power to the various components, and featured a variable resistor to control the LED intensity. The voltage read-out from the light-to-voltage converter was acquired in real-time using a microcontroller (Arduino Nano). Transmission of the data was either *via* an on-board Bluetooth module (Roving Networks RN-42) or USB wire from the microcontroller. The data was relayed to a LabView (National Instruments) virtual instrument written in-house to allow data processing and visualisation in real-time. In addition to transmitting the data, the microcontroller continuously saved the data down to a micro SD card so that no data would be lost in case of disconnection from the laptop. The saved data was later analysed offline with MATLAB (Mathworks) for detailed data processing and plotting.

### Absorbance measurement

For the in vitro testing the intensity (*I*) of the incident light at the light-to-voltage converter was converted to absorbance (*A*) according to the Beer–Lambert law

1 $$A = - \log _{10}\left( {\frac{{I_{{\mathrm{d}},{\mathrm{sample}}}}}{{I_{{\mathrm{d}},{\mathrm{blank}}}}}} \right){.}$$

Where *I*_d,blank_ and *I*_d,sample_ refers to the light intensity transmitted through droplets of blank and sample, respectively. For in vivo testing a modified version of the equation was used that also included terms for the intensity of the adjoining oil segments

2 $$A = - \log _{10}\left( {\frac{{I_{{\mathrm{d}},{\mathrm{sample}}} \times I_{{\mathrm{o}},{\mathrm{blank}}}}}{{I_{{\mathrm{d}},{\mathrm{blank}}} \times I_{{\mathrm{o}},{\mathrm{sample}}}}}} \right){.}$$

Where *I*_o,blank_ and *I*_o,sample_ refer to measurements conducted on the oil segments adjoining the blank and sample droplets being measured. As previously described^31^ these extra oil measurements correct for any drift in the intensity of the LEDs, as the absorbance of the oil will not change.

### Comparison of screw pump droplet generation to traditional coflow method

To compare droplet generation by the screw pump versus traditional passive droplet generation using syringe pumps (PHD 22/2000, Harvard Apparatus, UK), we first recorded droplet generation over 15 min using our pump (running at 0.35 Hz, 3 mm pitch) and then repeated the same experiment using fluid supplied by syringe pumps. The syringe pumps were fitted with 1 ml plastic syringes and delivered fluid at flow rates set to match the oil: aqueous flow ratio (1:1) at a similar total flow rate (measured to be 3.2 µL min^−1^, versus 2.5 µL min^−1^ for the screw-driven pump). The same microfluidic chip was used in each instance (Supplementary Fig. 10a, c). In each case data was collected from the moment the pumps were first started, and the two aqueous streams were composed of dyed and undyed deionised water such that the resulting droplet colour could be used as a measure of droplet composition (Fig. 2s). To characterise the generated droplets, a portable microscope camera (dnt Digimicro Mobile Mikroskope) was used to record the droplets once they have exited the chip into PTFE tubing of known cross-section. The videos recorded were analysed using droplet morphology and velocimetry software^68^, and the data subsequently processed in Matlab.

### In vitro glucose testing

To measure the difference in temporal performance between droplet and continuous microfluidics, a step-change in the incoming sample stream was achieved by alternating the pump’s dialysate (pull) line between solutions of 0 and 5 mM glucose in PB. To perform the assay in a continuous flow regime, the oil inlets were swapped with PB and the glucose concentration increased to 15 mM to compensate for the resulting dilution. The reagent concentration was not changed as it was already in excess. For both flow regimes the measurement point was approximately 60 mm downstream of the microfluidic chip and corresponded to a time lag of 3 min between droplet generation and measurement.

### In vivo glucose testing

A concentric microdialysis probe (fabricated in-house, 13 kDa MWCO) was implanted at a depth of 1 mm in the dermis of the volar aspect of the non-dominant forearm in each individual (Supplementary Fig. 12), and left for 1 h before being used to allow the early-phase inflammatory response to subside. This constitutes the minimum recommended wait time^69^, and is consistent with previous studies. Previous work has shown that the hyperaemia response associated with subcutaneous probe implantation in the forearm resolves within 60–120 min^70,71^, and post-implantation waiting times of 30 min to 1 h have been successfully used^23,43,72^. Moreover, the stable baseline periods observed in the sensor and standard microdialysis data (Fig. 4a, Supplementary Fig. 5 and Supplementary Note 2) are consistent with the inflammatory response having stabilised. Longer-timescale foreign body response has been shown to cause the probe recovery to drift over several days^73^, however, as the longest test conducted here was 3.5 h long, this effect was assumed to be negligible.

The sensor was calibrated for its glucose response immediately before connection to the microdialysis probe using 0.25, 0.5, 1.0, 2.5, 5.0 and 7.5 mM glucose standards in PB (4.5, 9.0, 18, 45, 90 and 135 mg dL^−1^). Sterilised PBS was used as the perfusate (pH 7.4, Tayside Pharmaceuticals, UK). All onboard fluids (oil, reagent, perfusate and waste) were housed in flexible plastic bags manufactured in-house by heat-sealing (Impulse sealer FS-100) thin-film polyethylene. During calibration, the perfusate flow rate was collected for ten minutes and then weighed to confirm the flow rate was approximately 1 µL min^−1^, consistent with laboratory testing. The data from the absorbance flow cell was relayed to a laptop for real-time data analysis and visualisation. During initial experiments, data transfer was via a USB wire connection. Following successful validation of the device later experiments utilised the Bluetooth module.

To validate the device data, glucose levels in venous blood and interstitial fluid sampled via standard microdialysis were recorded every 10 min. Venous blood (0.5 ml) was obtained following the insertion of a 22 g cannula into the antecubital fossa, while standard microdialysate samples were obtained by perfusing a second microdialysis probe inserted beside the probe attached to the sensor (Supplementary Fig. 12) using a syringe pump at 1 μL min^−1^, matching that of the sensor. Blood and microdialysate glucose levels were measured using a commercially available glucose metre (Accu-Chek^®^ Aviva, Roche, UK). Blood and dialysate glucose levels were also independently measured by the Chemical Pathology laboratory at University Hospital Southampton NHS Foundation Trust. The results were in excellent agreement as shown in see Supplementary Fig. 13.

### Estimation of interstitial fluid glucose concentration

The glucose concentration in the tissue interstitial fluid was estimated using a two-point calibration method similar to those previously described for electrochemical CGM sensors^38,39^. This method assumes that interstitial fluid concentrations show similar concentrations and excursions to the blood glucose levels (albeit with a time lag), which is typically observed in healthy subjects^35,74^. A similar estimation technique is employed by the commercially available Menarini Glucoday sensor (which uses microdialysis and an electrochemical sensor in the dialysate stream) and does not measure in vivo probe recovery^23^.

The interstitial fluid concentration was derived using the following equation

3 $$\left[ G \right]_{{\mathrm{IF}}} = \left( {\left[ G \right]_{\mathrm{D}} - \left[ G \right]_{{\mathrm{D}},0}} \right) \times \frac{{\Delta [G]_{\mathrm{B}}}}{{\Delta [G]_{\mathrm{D}}}} + [G]_{{\mathrm{B}},0}{,}$$

where [*G*]_IF_ is the estimated glucose concentration in the interstitial fluid, [*G*]_D_ is the measured glucose concentration in the dialysate, [*G*]_D,0_ is the baseline dialysate glucose concentration, Δ[*G*]_B_ is the difference between the peak and baseline blood glucose concentrations, Δ[*G*]_D_ is the difference between the peak and baseline dialysate glucose concentrations and [*G*]_B,0_ is the baseline blood glucose concentration.

### In vivo lactate testing

As for the glucose testing, two concentric microdialysis probes (fabricated in-house, 13 kDa MWCO) were inserted into the upper dermis of the volar aspect of the forearm, for attachment of the sensor and additional sampling via standard microdialysis. A 22 g cannula was inserted into the antecubital fossa for collection of venous blood samples. Lactate levels in blood and standard microdialysate were measured using a commercially available lactate metre (Arkray Lactate Pro2). We found that the lactate metre gave consistently high readings when measuring dialysate. To address this, a calibration curve was formed using the same standards used to calibrate the sensor (which like the dialysate, used phosphate buffer as the medium) and used to correct all standard microdialysis results. To ensure accuracy, the standards were independently checked by the Critical Care Laboratory at University Hospital Southampton NHS Foundation Trust, and were in good agreement with their nominal values as shown in Supplementary Fig. 14.

At the start of the experiment, lactate levels were continuously monitored for approximately 20–30 min to establish a baseline, with blood and standard microdialysate measured every 5 min The blood pressure cuff placed around the upper arm was then inflated (pressure > 140 mmHg). During this period, the skin of the lower arm became visibly paler and mottled, consistent with a local ischaemia. The cuff was released after 5 min blood and standard microdialysate levels were then measured at an increased rate of every 2 min, for a period of 10 min. The sampling frequency then returned to its initial rate of every 5 min. Following another baseline period the cuff inflation was repeated. This study was conducted in four individuals.

### Ethics and safety

The in vivo testing in healthy volunteers was performed following review and approval by the Faculty of Health Sciences Ethics Committee, University of Southampton (FOHS 9349), and informed and signed consents were obtained from all participants. The device was designed to meet medical electrical safety requirement and featured no electrical contact between the pump, optical components and the sample stream. The tubing isolated the liquid sample path from any electronic components, while the tubing was further isolated from all optical components by the body of the acrylic flow cell. The device is battery powered at a low voltage of 1.5 V, therefore, the whole device (including the microdialysis probe) effectively functions as a Class III device under IEC 60601.

### Modelling of glucose data

Prediction of the variation in blood glucose from the sensor data was calculated using a model previously reported by Steil and co-workers^37,38^. This model treats the system as two compartments, blood and interstitial fluid, with volumes *V*_1_ and *V*_2_ and glucose concentrations [*G*]_1_ and [*G*]_2_ respectively (as illustrated in Supplementary Fig. 15). Glucose transfer from blood to glucose, and vice versa, is described by rate constants *k*_21_ and *k*_12_, whilst constant *k*_02_ describes the transfer from interstitial fluid to neighbouring cells. The mass balance equation characterising the movement of glucose is given by

4 $$\frac{{dV_2\left[ G \right]_2}}{{dt}} = k_{21}V_1\left[ G \right]_1 - \left( {k_{12} + k_{02}} \right)V_2[G]_2{.}$$

As the volumes and rate constants cannot be experimentally determined, Eq. (4) must be modified to include parameters that can be. As described in detail by Steil and coworkers^37,38^, it can be rearranged to

5 $$\frac{{d\left[ G \right]_2}}{{dt}} = p_1\left[ G \right]_1 - p_2\left[ G \right]_2{,}$$

where $$p_1 = k_{21}\frac{{V_1}}{{V_2}}$$ and *p*_2_=(*k*_12_+*k*_02_).

*p*_1_/*p*_2_ corresponds to the blood plasma—interstitial fluid glucose gradient, which under steady-state baseline conditions will correspond to

6 $$\left[ G \right]_{2,{\mathrm{b}}} = \frac{{p_1}}{{p_2}}[G]_{1,{\mathrm{b}}}{,}$$

where [*G*]_1,b_ and [*G*]_2,b_ correspond to the glucose concentrations in the blood and interstitial fluid under baseline conditions. The interstitial fluid glucose, [*G*]_2_ is not directly measured in our experiments, however, as the sensor measures the concentration in the dialysate, which is related to interstitial fluid concentration by the relative recovery

7 $$\left[ G \right]_{\mathrm{d}} = R\left[ G \right]_2{,}$$

where [*G*]_d_ is the dialysate glucose concentration, as measured by the sensor and *R* is the relative recovery. We assume here that the glucose exchange dynamics across the microdialysis probe membrane are much faster than the tissue dynamics and hence negligible. Substituting (7) into (5) and (6) gives

8 $$\frac{{d\left[ G \right]_{\mathrm{d}}}}{{dt}} = p_3\left[ G \right]_1 - p_2\left[ G \right]_{\mathrm{d}}{,}$$

9 $$\left[ G \right]_{{\mathrm{d}},{\mathrm{b}}} = \frac{{p_3}}{{p_2}}[G]_{1,{\mathrm{b}}}{,}$$

where *p*_3_ = *Rp*_1_.

*p*_2_ can be experimentally determined as it corresponds to the blood–interstitial fluid delay time (formally, 1/*p*_2_ is defined as the time to reach 63% equilibration)^38^. Once *p*_2_ has been determined, *p*_3_ can be determined from the baseline measurements using Eq. (9).

The blood glucose was calculated from the sensor data by rearranging Eq. (8) for [*G*]_1_. As previously highlighted^37^, the presence of a derivative (*d*[*G*]_d_/*dt*) means that the calculated values are inherently noisy and smoothing is required. Due to the high measurement frequency of our sensor, this could be easily achieved using a simple rolling average algorithm.

### Reporting summary

Further information on research design is available in the Nature Research Reporting Summary linked to this article.

## Supplementary information

Supplementary Information

Reporting Summary

Description of Additional Supplementary Files

Supplementary Movie 1

Supplementary Movie 2

Supplementary Movie 3

## Data Availability

The datasets generated during and/or analysed during the current study are available from the corresponding author on reasonable request.
