# Supplementary material for: Monitoring biomolecule concentrations in tissue using a wearable droplet microfluidic-based sensor
Source: Nat Commun. 2019 Jun 21;10:2741. doi: 10.1038/s41467-019-10401-y (PMC6588579; doi:10.1038/s41467-019-10401-y)
Supplement: Supplementary file 1 — Supplementary Information [file 41467_2019_10401_MOESM1_ESM.pdf]

Supplementary information for

Monitoring biomolecule concentrations in tissue using a wearable droplet microfluidic-based sensor

Adrian M. Nightingale,[a] Chi Leng Leong,[a,b] Rachel A. Burnish,[c] Sammer-ul Hassan,[a] Yu Zhang,[a] Geraldine F. Clough,[d] Martyn G. Boutelle,[b] David Voegeli,[e] and Xize Niu[a†]

a. Faculty of Engineering and Physical Sciences, University of Southampton, Southampton, U.K. SO17 1BJ.

b. Department of Bioengineering, Imperial College London, South Kensington, London, U.K. SW7 2AZ

c. Critical Care/ Anaesthesia and Perioperative Medicine Research Unit, University Hospital Southampton NHS Foundation Trust, Tremona Road, Southampton, U.K. SO16 6YD

d. Faculty of Medicine, University of Southampton, Southampton, U.K. SO17 1BJ

e. Faculty of Environmental and Life Sciences, University of Southampton, Southampton, U.K. SO17 1BJ. Now at Department of Sport, Exercise & Health, University of Winchester, Winchester, UK. SO22 4NR

† Email: x.niu@soton.ac.uk

Supplementary figures

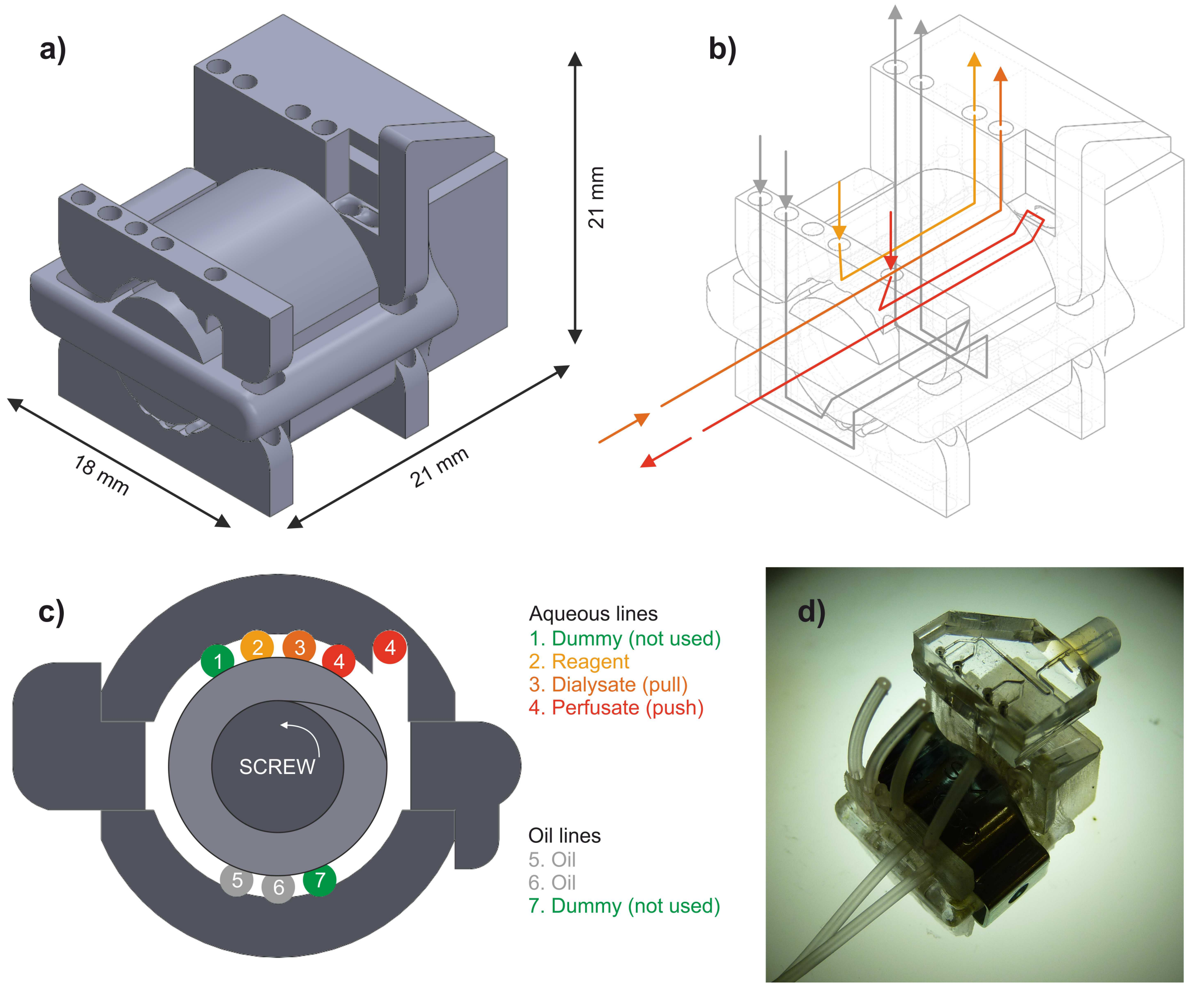

Supplementary Figure 1 | Images showing the pumphead used for glucose measurement. a) Rendering of the 3D printed parts of the pump-head. b) Schematic showing how tubing was threaded through the pumphead, with tubing running from the front of the pump to the back, positioned parallel to the axis of the screw. The pathway of fluid is highlighted by arrows. The dialysate and perfusate lines are orientated at the front of the pump head to allow easy connection of the microdialysis probe. c) Cross-sectional view of the pumphead showing the relative positions of the pumplines around the screw. The oil lines are located at the bottom of the screw while the aqueous lines are at the top, ensuring the pulses of the oil and aqueous flows are out of phase. The dummy lines (1,7, coloured green) are not used but are included to ensure the pressure on the screw is symmetrical. The perfusate line (4, coloured red), in contrast to the other aqueous lines, returns to the front of the pump via a recessed channel. d) Image of an assembled pump, including clip to ensure the tubing compresses the screw, and PDMS microfluidic chip at the exit point.

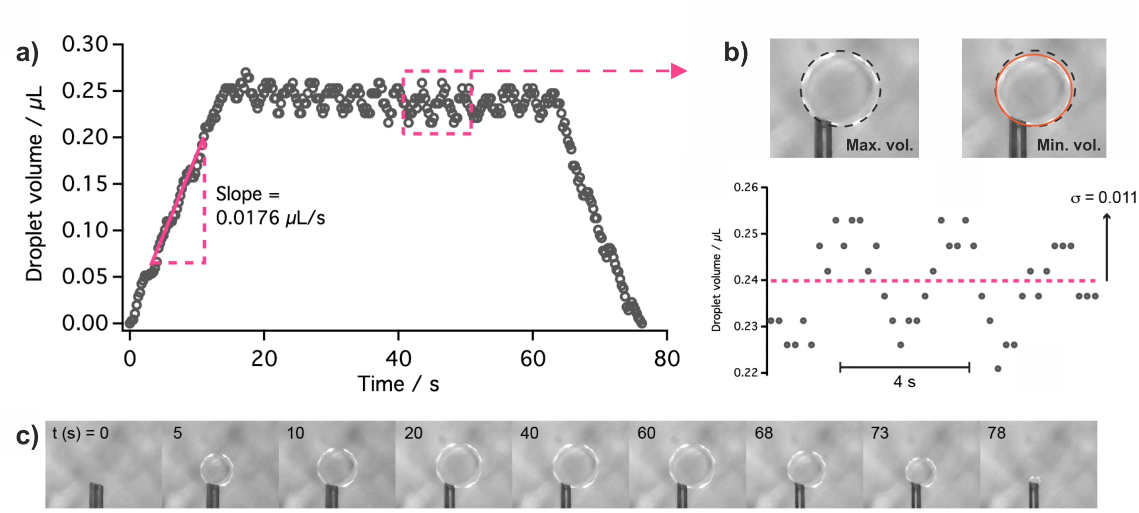

Supplementary Figure 2 | Demonstrating push/pull pump operation. a) Droplet volume over time during the experiment. The droplet volume rose when the push line alone was connected, stabilised with the connection of the pull line (14 s) and then decreased when the push line was disconnected (64 s). b) At equilibrium, i.e. pushing and pulling at the same rate, there was a slight oscillation of droplet volume. The droplet pulsed from a maximum to a minimum volume due to the turning of the motor and the relative position of the fluid lines on the screw thread. c) Representative images of the droplet during the experiment.

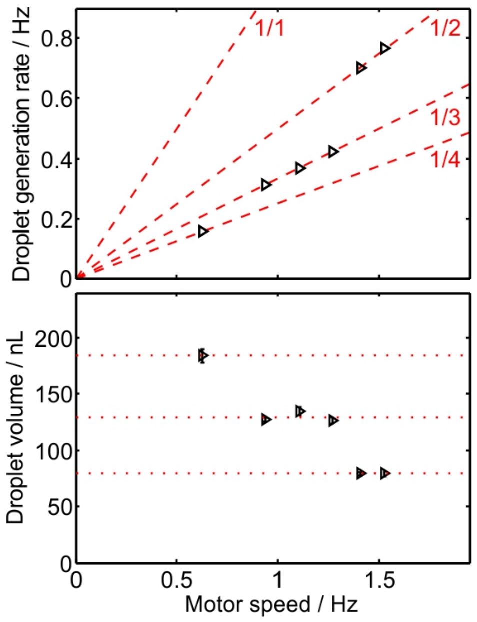

Supplementary Figure 3 | Droplet generation dynamics using a 2 mm pitch screw. One droplet is generated in every two, three or four turns of the motor depending on motor speed (top), with a commensurate change in mean droplet volume (bottom). Error bars give the standard deviation in droplet size (n > 30).

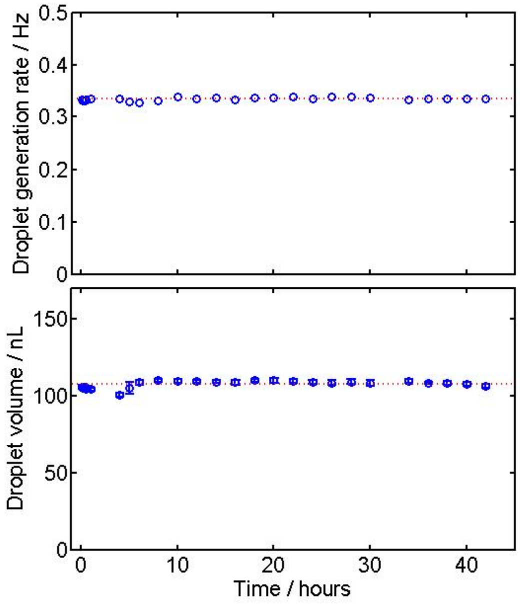

Supplementary Figure 4 | Droplet generation with the screw-driven pump running at steady-state. Over the course of 42 hours droplet generation rate (top) and mean volume (bottom) remained constant. Error bars give the standard deviation in droplet size (n > 30).

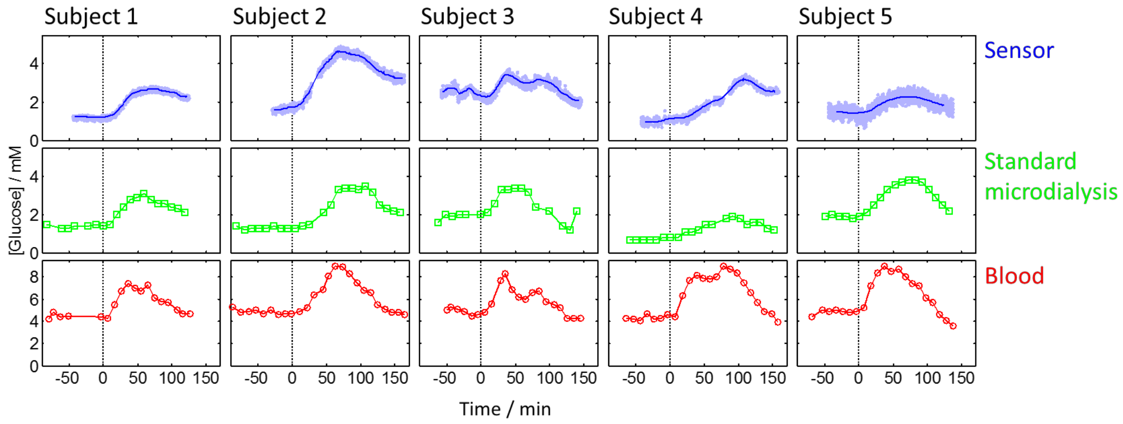

Supplementary Figure 5 | Comparison of glucose measured in interstitial fluid and blood. Glucose concentrations for microdialysate measured by our device (top row), microdialysate obtained by standard microdialysis and measured with a point-of-care glucometer (middle row) and blood glucose measured with point-of-care glucometer (bottom row). The data are shown for each individual subject (columns 1-5).

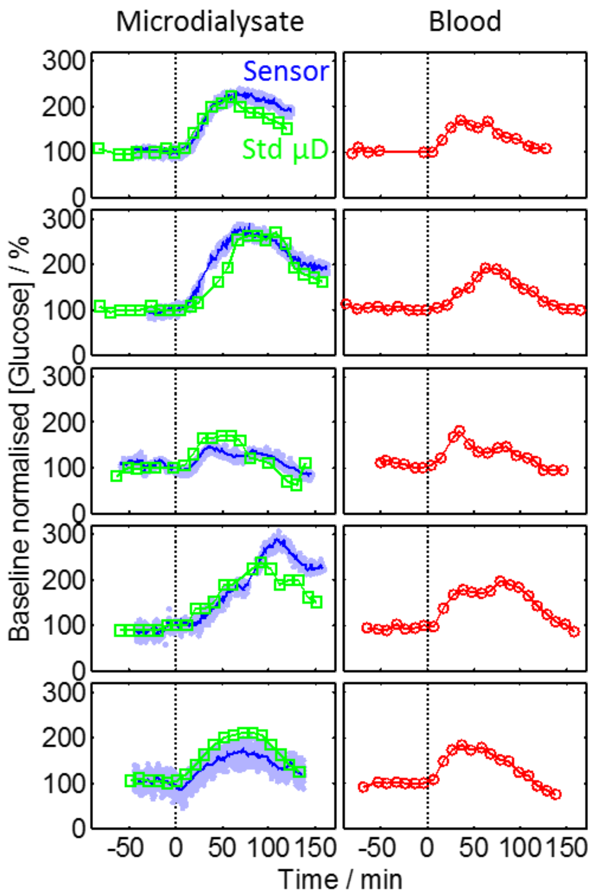

Supplementary Figure 6 | Deviations from baseline levels for glucose in interstitial fluid and blood. Glucose concentrations are normalised to baseline levels for microdialysate (left column) and blood (right column). The microdialysate column shows data obtained from our sensor (light blue data points and dark blue line) and standard microdialysis and offline analysis (green squares). The data are shown for each individual subject (rows 1-5).

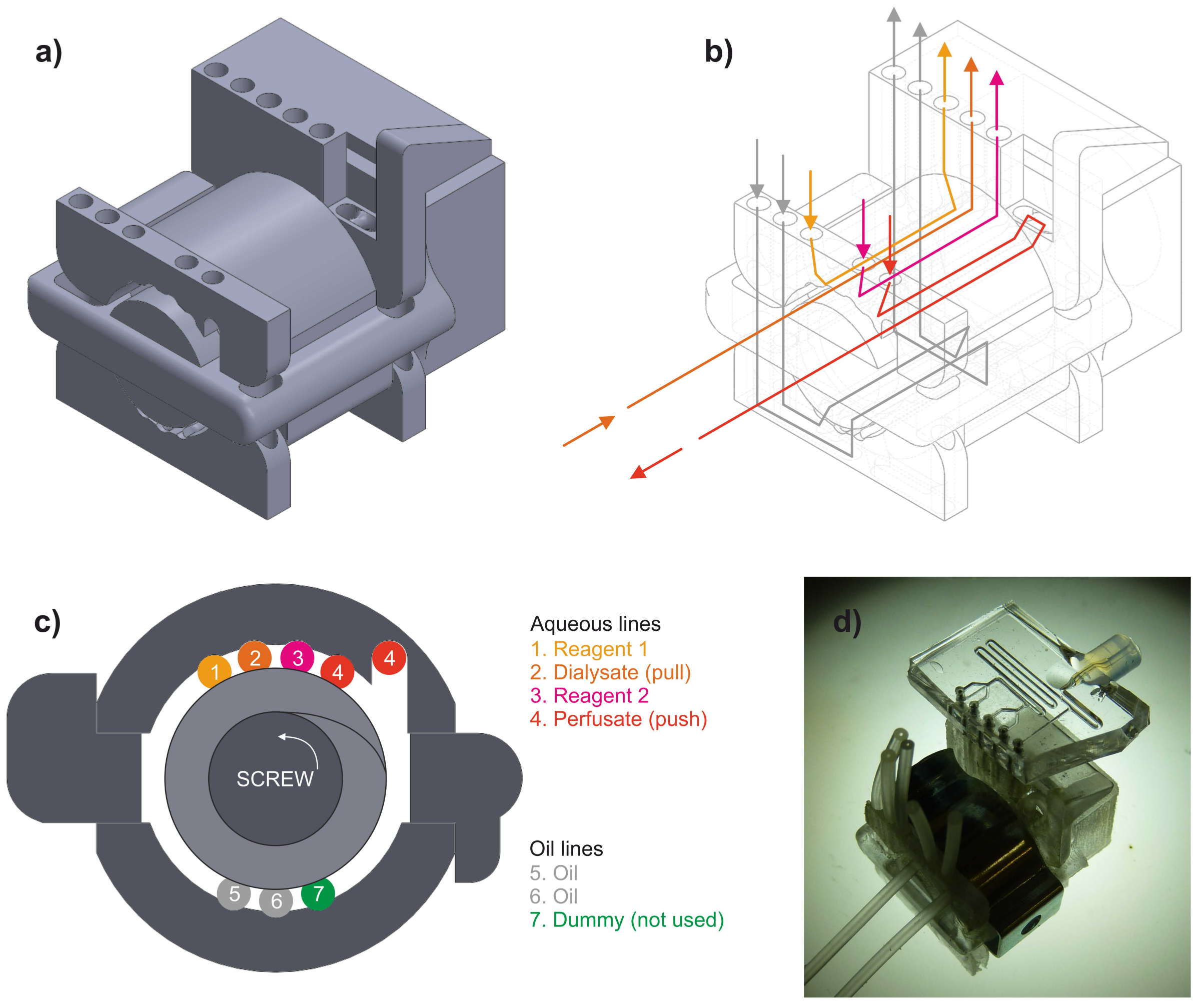

Supplementary Figure 7 | Images showing the pumphead used for lactate measurement. In contrast to the glucose pump shown in Supplementary Figure 1, this design includes an additional aqueous line. a) Rendering of the 3D printed parts of the pump-head. b) Schematic showing how tubing was threaded through the pumphead, with tubing running from the front of the pump to the back, positioned parallel to the axis of the screw. The pathway of fluid is highlighted by arrows. The dialysate and perfusate lines are orientated at the front of the pump head to allow easy connection of the microdialysis probe. c) Cross-sectional view of the pumphead showing the relative positions of the pumplines around the screw. The oil lines are located at the bottom of the screw while the aqueous lines are at the top, ensuring the pulses of the oil and aqueous flows are out of phase. The dummy line (7, coloured green) is not used but is included to ensure the pressure on the screw is symmetrical. The perfusate line (4, coloured red), in contrast to the other aqueous lines, returns to the front of the pump via a recessed channel. d) Image of an assembled pump, including clip to ensure the tubing compresses the screw, and PDMS microfluidic chip at the exit point.

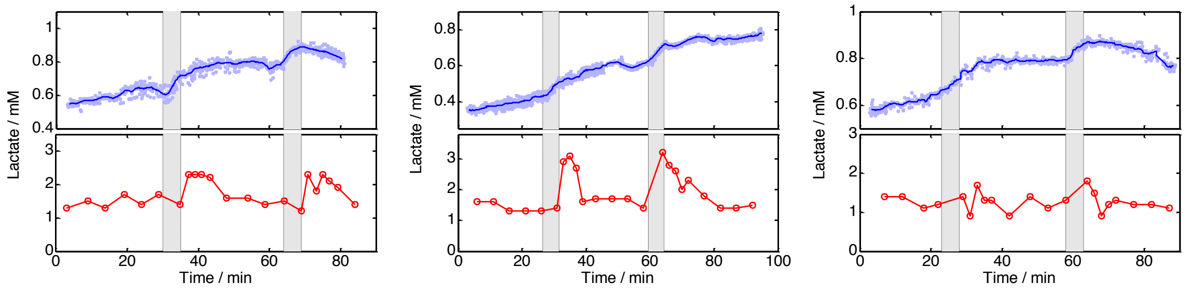

Supplementary Figure 8 | In vivo lactate measurement. Measurements from three experiments supplementary to the experimental data shown in the main text. In each case, the sensor measurement of dialysate from subcutaneous interstitial fluid is shown top. Each blue data point represents a measurement from a single droplet with the line indicated the running average. The shaded areas indicate periods of blood occlusion to the tissue. Shown bottom is the blood lactate during the same period. As discussed in the main text, each occlusion period results in an increase in tissue lactate, with a short-lived increase in blood lactate (of varying magnitude) immediately following the occlusion.

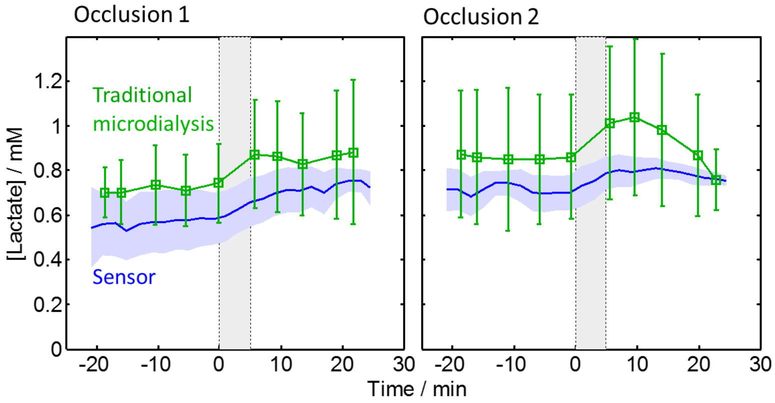

Supplementary Figure 9 | Comparison of lactate measured by the sensor and traditional microdialysis. Average (n = 4) dialysate lactate measured by the sensor (blue line) and standard microdialysis sampling and offline analysis (green squares and error bars) for each of the two occlusions performed in each test. Each is shown as a function of time relative to the start of occlusion, and the occlusion period is highlighted by the grey shaded areas between t = 0 and t = 5. The green error bars and light blue shaded region correspond to the standard deviation from subject-to-subject variation.

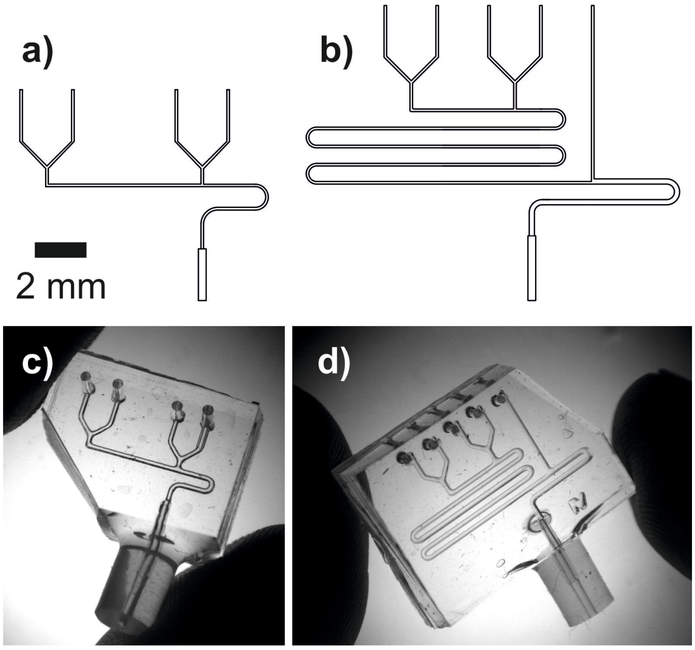

Supplementary Figure 10 | Microfluidic chip schematics and images. a) A schematic of the glucose microfluidic chip. b) A schematic of the design of the lactate chip used for all in vivo experiments. Note the wider section after the addition of the second reagent is shorter than that used for laboratory testing (see Fig. 5b) as the time required for reaction two to complete (see Supplementary Figure 7e and accompanying discussion) is short enough that the extra channel length is not required. c) A finished glucose chip held between thumb and forefinger. d) A finished lactate chip held between thumb and forefinger.

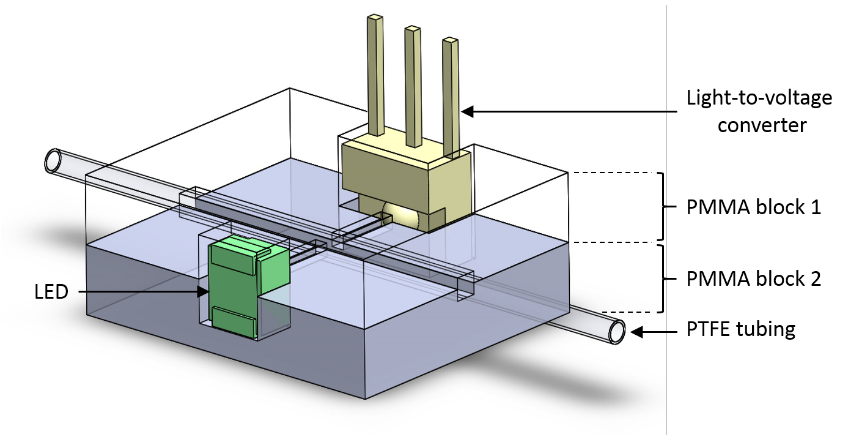

Supplementary Figure 11 | 3D schematic of the flow cell. An LED, light-to-voltage converter and PTFE tubing are positioned using a micromilled PMMA substrate micromilled from two separate blocks. Here PMMA block 1 is shown transparent, however in real-life all PMMA was tinted black so that light could only pass through the micromilled channel orthogonal to the main PTFE tubing groove.

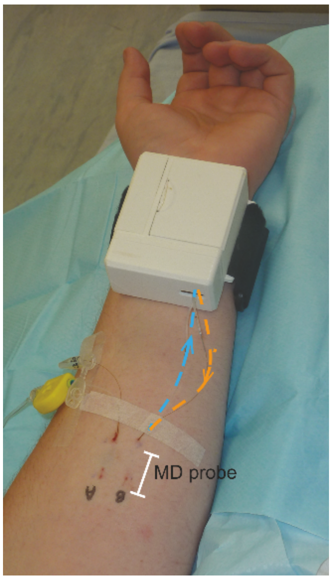

Supplementary Figure 12 | Sensor attached to microdialysis probe inserted into forearm. The probe and the fluid path from the sensor to/from the probe are highlighted.

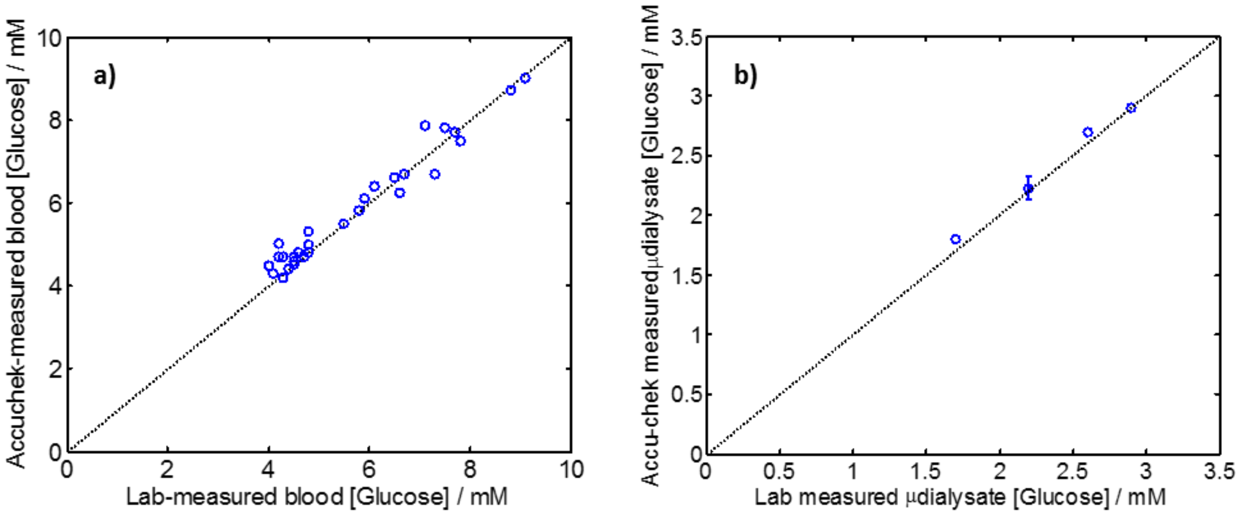

Supplementary Figure 13 | Cross-checking analytical methods for offline glucose analysis. Comparison of Accu-chek derived glucose concentrations for blood (a) and microdialysate (b) relative to values obtained from the pathology laboratory at Southampton General Hospital. The dashed lines are of unity gradient, indicating the values expected for exact equivalency. The error bar in (b) shows the standard deviation for a measurement repeated 5 times.

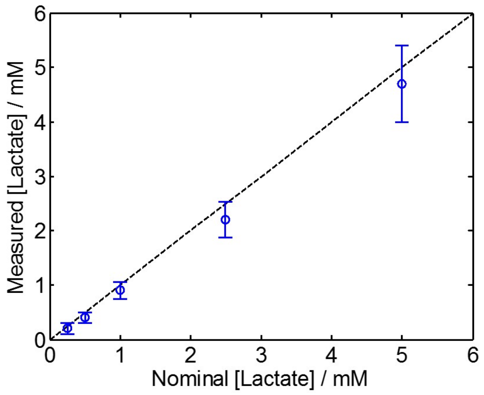

Supplementary Figure 14 | Checking concentrations of lactate standards. Analysis of the lactate standards used to calibrate our sensor and the point-of-care device used to measure standard microdialysate. Analysis was carried out by the Critical Care Laboratory at Southampton General Hospital using a Radiometer ABL800 Flex blood gas analyser. Error bars correspond to the measurement variance as detailed in the analyser's manual.

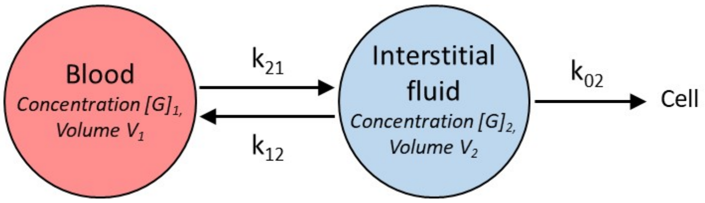

Supplementary Figure 15 | Schematic illustrating the two-compartment glucose model. All parameters used to describe the movement of glucose between the blood and interstitial fluid, described in the Methods section of the main text, are also shown.

Supplementary notes

Supplementary note 1

To demonstrate the simultaneous pushing and pulling actions of the pump, we carried out an experiment using a push-pull probe. The probe, fabricated in-house, consisted of two pieces of fused silica capillary (ID 75 µm, OD 150 µm), glued together, cut and polished such that both tips were flush.

We connected the inlet and outlet of the push-push probe to the perfusate (pushing) and dialysate (pulling) lines of the pump respectively. The perfusion fluid or perfusate was deionized water. The tip of the probe was immersed in a cuvette of FC-40 oil. A USB camera was used to monitor the change in volume of a droplet generated at the probe tip submerged in FC-40 oil. The video was converted into grey-scale image stack and analysed frame-by-frame using ImageJ. The results are shown in Supplementary Figure 2 and Supplementary Movie 1.

Initially, only the push (perfusate) line was connected to allow an aqueous droplet to form and grow at the tip of the probe. After approximately 14 s, the pull line was connected and the aqueous droplet was subsequently observed to remain constant in size, with a slight pulsation – expanding and contracting as shown in Supplementary Figure 2b and Supplementary Movie 1. The pulsation was a visualisation of the slightly offset pushing and pulling actions of the pump at equilibrium. The pulsation phase was stable, with a time period of approximately three seconds. After 50 seconds the push line was disconnected and the droplet drawn into the pulling arm of the probe. From the rising and falling slopes of Supplementary Figure 2a, the flow rate was found to be 1.06 ± 0.06 µL min^{-1}, which was in agreement with the flow rate later measured during clinical microdialysis.

Supplementary note 2

Supplementary Figure 5 compares the glucose concentration in the dialysate measured by the sensor (top row), standard microdialysis (centre row), and blood (bottom row) for each of the five subjects. Note, the standard microdialysis results for subject 2 and 3 show a plateau region at the upper limit suggestive of a measurement ceiling ("limiting out"), however the measurement was well within the operational range of the glucometer and we have no reason to suspect these plateau regions are measurement artefact.

While the baseline blood levels are relatively uniform at~4.5 mM, the baseline levels of the sensor and standard microdialysis results vary. Subject 2, for example, had baseline values of 1.7 and 1.3 mM for sensor and standard microdialysis respectively, while subject 5 had baseline values of 1.4 and 1.9 mM respectively. The greater variation in the microdialysis-based tissue measurements when compared to the blood concentrations is also apparent by comparing the standard deviations during the baseline periods shown in Fig.s 4a,b. The variation in baseline levels in both the sensor and standard microdialysis is consistent with the previous reports showing probe recovery varies with the exact

location of implantation[1,2] due to the glucose mass transport being dependent on the extracellular channels surrounding the probe and the proximity of local vasculature. To test this hypothesis we plotted the sensor and standard microdialysis results normalised to the baseline levels (Supplementary Figure 6, left). Here the excursions following oral glucose administration were of very similar magnitude and timing, especially when compared to the equivalent blood results (Supplementary Figure 6, right). This is consistent with the variations in absolute concentrations (Supplementary Figure 5) being due to the *in vivo* probe recovery varying on a case-by-case basis and not due to any fundamental difference between the sensor and the standard microdialysis. We would expect this variation to average out over several subjects and this was found to be the case, as shown in Fig. 4a.

Supplementary note 3

Supplementary Figure 8 compares the variation of lactate concentrations as measured in blood (red circles and line) and in subcutaneous tissue by the sensor (light blue points and dark blue line) for three individual volunteers. Note, the sensor values represent the concentrations measured in the dialysate. The tissue was subjected to two periods of mild ischaemia (grey shaded regions, see Methods). The sensor values are in the expected range,[3,4] and lower than the actual concentrations in the interstitial fluid due to the *in vivo* recovery of the probe.[5] The results in Supplementary Figure 8 are additional to the set of example results shown in the main manuscript (Fig. 5d,e).

It has been previously shown that general trends in blood and tissue lactate are not directly comparable,[6] however the effect of the ischaemia here is evident in both the observed blood and tissue lactate concentrations. Basal blood lactate remained relatively constant, rapidly increased following occlusion and then returned to basal levels within ten minutes as lactate was cleared. In comparison, the subcutaneous interstitial lactate generally showed a slight upward trend (possibly due to glycolytic stimulation from probe insertion) which was augmented during the ischaemic periods. In the example data shown in the main text, (Fig. 5d,e) the subcutaneous lactate levels showed the opposite trend under steady state conditions (a slow decrease) however that was in the context of overall decreasing blood levels (final blood reading ~28% of first) as opposed to the approximately constant blood baseline here. Overall the observed concentrations[3,4] and trends[7] are consistent with previous studies using microdialysis to measure tissue lactate.

Supplementary references

1 Brunner, M. *et al.* Validation of urea as an endogenous reference compound for the in vivo calibration of microdialysis probes. *Life Sci.* **67**, 977-984, (2000).

2 de Lange, E. C. M., Danhof, M., de Boer, A. G. & Breimer, D. D. Methodological considerations of intracerebral microdialysis in pharmacokinetic studies on drug transport across the blood–brain barrier. *Brain Res. Rev.* **25**, 27-49, (1997).

3 Ellmerer, M. *et al.* Continuous measurement of subcutaneous lactate concentration during exercise by combining open-flow microperfusion and thin-film lactate sensors. *Biosens. Bioelectron.* **13**, 1007-1013, (1998).

4 Rosdahl, H., Ungerstedt, U., Jorfeldt, L. & Henriksson, J. Interstitial glucose and lactate balance in human skeletal-muscle and adipose-tissue studied by microdialysis. *J. Physiol.* **471**, 637-657, (1993).

5 Chefer, V. I., Thompson, A. C., Zapata, A. & Shippenberg, T. S. Overview of Brain Microdialysis. *Current protocols in neuroscience / editorial board, Jacqueline N. Crawley ... [et al.]*, Unit7.1-Unit7.1, (2009).

6 Ellmerer, M. *et al.* Clinical Evaluation of Subcutaneous Lactate Measurement in Patients after Major Cardiac Surgery. *Int. J. Endocrinol.*, (2009).

7 Korth, U. *et al.* Tourniquet-induced changes of energy metabolism in human skeletal muscle monitored by microdialysis. *Anesthesiology* **93**, 1407-1412, (2000).
