# Supplementary material for: Monitoring biomolecule concentrations in tissue using a wearable droplet microfluidic-based sensor
Source: Nat Commun. 2019 Jun 21;10:2741. doi: 10.1038/s41467-019-10401-y (PMC6588579; doi:10.1038/s41467-019-10401-y)
Supplement: Supplementary file 3 — Description of Additional Supplementary Files [file 41467_2019_10401_MOESM3_ESM.docx]

**Description of Additional Supplementary Files**

File Name: Supplementary Movie 1

Description: Push/pull pumping demonstration

File Name: Supplementary Movie 2

Description: Droplet generation under standard conditions using microfluidic chip for glucose measurement

File Name: Supplementary Movie 3

Description: Two-step lactate assay performed on-chip. Colour development only occurs after dosing of the second reagent
